# P-652. Development of a Severity Scoring System for Mycoplasma pneumoniae Infection

**DOI:** 10.1093/ofid/ofaf695.865

**Published:** 2026-01-11

**Authors:** Kristen Kelly, Lilly Cheng-Immergluck, Declan Quinn

**Affiliations:** University of Chicago Comer Children's Hospital, Chicago, IL; University of Chicago, Chicago, Illinois; University of Chicago, Chicago, Illinois

## Abstract

**Background:**

*Mycoplasma pneumoniae* (MP) causes ∼2 million infections in the U.S.[1] with increasing rates reported globally in the past few years [2-4]. Rates in children have increased from 1% to 7.2% among age 2-4 and from 3.6% to 7.4% in those 5 -17yo [1]. In Illinois, MP rates are about 5.1%, which is higher than rates of RSV, Flu or Covid [3]. While MP usually presents as “walking pneumonia” in school-aged children, there have been increases in extrapulmonary manifestations. We propose a severity scoring system for MP based off of the Westley Croup Score (WCS) and Phoenix Sepsis Criteria (PSC). Both have assessments of neurologic status. The WCS assesses degrees of respiratory distress. The PSC assesses vitals and state of inflammation.

**Figure 1.**
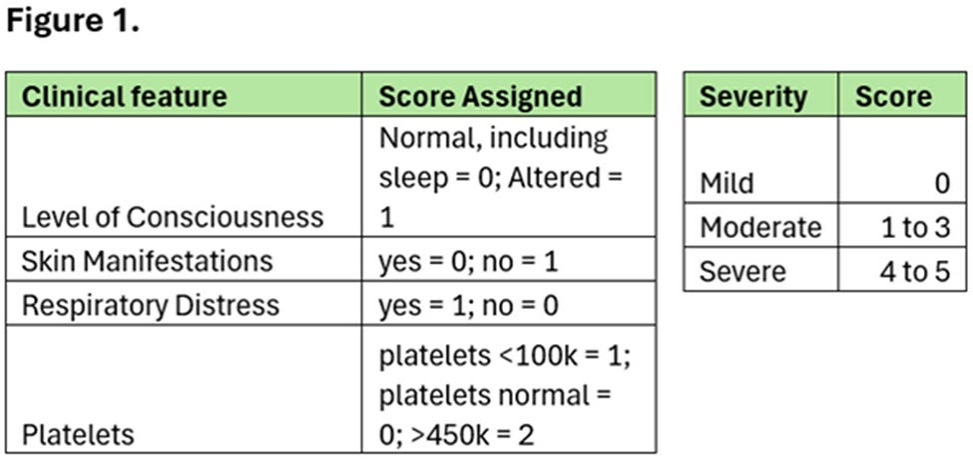
The Mycoplasma Pneumoniae Severity Score (MPSS)

The MPSS consists of components of the validated WSC and the PSC. MPSS combines components of vital abnormalities, respiratory distress, mental status, skin manifestations and lab findings to predict the severity of illness of MP.

**Methods:**

This is a retrospective analysis of data collected from a network of healthcare systems in Chicago, which are a part of the NIH-funded Institute of Translational Medicine. Data was collected from 2014-2024 and included data on patient demographics, dates of infection, clinical symptoms, and disease severity were captured from electronic health records. Severity scores were created and assigned based on the adapted MP severity score (MPSS) constructed from the WCS and PSC. Statistical analyses include χ^2^ tests or *t* tests to determine the association between MP and potential risk factors. Multilevel model includes patient level data and area level covariates, which are based on proxies identified as risks factors. We will apply a generalized estimated equations (GEE) model; and crude odds ratios (OR) will be based on conditions determined *a priori* to be associated with risk of MP and used as estimates of relative risks. All tests for significance is two-tailed, and a *p*-value of < 0.05 is considered significant.

**Results:**

Since 2014, there have been 82 MP infections at UCM for which 19 (23%) were hospitalized: 35 (43%) of these infections from 1/2024 to 12/2024 compared to 2 non-admitted infections during the COVID19 pandemic (2020-2022). In the last 10 years, there have only been 11 infections in < 6 years of age (all in 2024). 19 patients were hospitalized. 8 were found to be mild, 9 were moderate and 2 were severe.

**Conclusion:**

More surveillance is needed in the United States to determine how prevalent and severe MP is in our pediatric population now and into the future.

**Disclosures:**

Lilly Cheng-Immergluck, MD, MS, American Academy of Pediatrics: Board Member|Department of Energy: Grant/Research Support|National Institutes of Health: Grant/Research Support|Pfizer: Grant/Research Support|Sanofi: Grant/Research Support

